# First trimester use of artemisinin-based combination therapy and the risk of low birth weight and small for gestational age

**DOI:** 10.1186/s12936-020-03210-y

**Published:** 2020-04-08

**Authors:** Orvalho Augusto, Andy Stergachis, Stephanie Dellicour, Halidou Tinto, Anifa Valá, Maria Ruperez, Eusébio Macete, Seydou Nakanabo-Diallo, Adama Kazienga, Innocent Valéa, Umberto d’Alessandro, Feiko O. ter Kuile, Gregory S. Calip, Peter Ouma, Meghna Desai, Esperança Sevene

**Affiliations:** 1 Department of Global Health, School of Public Health, University of Washington, Seattle, USA; 2 Faculty of Medicine, Eduardo Mondlane University, Maputo, Mozambique; 3 Centro de Investigação em Saúde da Manhiça, Manhiça, Mozambique; 4 Department of Pharmacy, School of Pharmacy, University of Washington, Seattle, USA; 5 Department of Clinical Medicine, Liverpool School of Tropical Medicine, Liverpool, UK; 6 Institut de Recherche en Sciences de la Santé/URCN, Nanoro, Burkina Faso; 7 Barcelona Institute of Global Health, University of Barcelona, Barcelona, Spain; 8 Medical Research Council Unit The Gambia at the London School of Hygiene and Tropical Medicine, Fajara, The Gambia; 9 Department of Pharmacy Systems, Outcomes and Policy, University of Illinois at Chicago, Chicago, IL USA; 10 Kenya Medical Research Institute Centre for Global Health Research, Kisumu, Kenya; 11 Centers for Disease Control and Prevention, Atlanta, GA USA

**Keywords:** Low birth weight, Small for gestational age, Prospective cohort, Artemisinins, Sub-Saharan Africa, Pharmacovigilance

## Abstract

**Background:**

While there is increasing evidence on the safety of artemisinin-based combination therapy (ACT) for the case management of malaria in early pregnancy, little is known about the association between exposure to ACT during the first trimester and the effect on fetal growth.

**Methods:**

Data were analysed from prospective studies of pregnant women enrolled in Mozambique, Burkina Faso and Kenya designed to determine the association between anti-malarial drug exposure in the first trimester and pregnancy outcomes, including low birth weight (LBW) and small for gestational age (SGA). Exposure to anti-malarial drugs was ascertained retrospectively by record linkage using a combination of data collected from antenatal and adult outpatient clinic registries, prescription records and self-reported medication usage by the women. Site-level data synthesis (fixed effects and random effects) was conducted as well as individual-level analysis (fixed effects by site).

**Results:**

Overall, 1915 newborns were included with 92 and 26 exposed to ACT (artemether–lumefantrine) and quinine, respectively. In Burkina Faso, Mozambique and Kenya at recruitment, the mean age (standard deviation) was 27.1 (6.6), 24.2 (6.2) and 25.7 (6.5) years, and the mean gestational age was 24.0 (6.2), 21.2 (5.7) and 17.9 (10.2) weeks, respectively. The LBW prevalence among newborns born to women exposed to ACT and quinine (QNN) during the first trimester was 10/92 (10.9%) and 7/26 (26.9%), respectively, compared to 9.5% (171/1797) among women unexposed to any anti-malarials during pregnancy. Compared to those unexposed to anti-malarials, ACT and QNN exposed women had the pooled LBW prevalence ratio (PR) of 1.13 (95% confidence interval (CI) 0.62–2.05, p-value 0.700) and 2.03 (95% CI 1.09–3.78, p-value 0.027), respectively. Compared to those unexposed to anti-malarials ACT and QNN-exposed women had the pooled SGA PR of 0.85 (95% CI 0.50–1.44, p-value 0.543) and 1.41 (95% CI 0.71–2.77, p-value 0.322), respectively. Whereas compared to ACT-exposed, the QNN-exposed had a PR of 2.14 (95% CI 0.78–5.89, p-value 0.142) for LBW and 8.60 (95% CI 1.29–57.6, p-value 0.027) for SGA. The level of between sites heterogeneity was moderate to high.

**Conclusion:**

ACT exposure during the first trimester was not associated with an increased occurrence of LBW or SGA. However, the data suggest a higher prevalence of LBW and SGA for children born to QNN-exposed pregnancies. The findings support the use of ACT (artemether–lumefantrine) for the treatment of uncomplicated malaria during the first trimester of pregnancy.

## Background

Malaria in pregnancy is an important public health problem in malaria-endemic countries where pregnant women and their offspring have a higher risk of infection and sequelae. Malaria infection during pregnancy is associated with maternal anaemia and intra-uterine growth restriction (IUGR), leading to poor pregnancy outcomes such as low birth weight (LBW) and small for gestational age (SGA) [1, 2]. LBW is defined as a birth weight of live-born infant of less than 2500 g regardless of gestational age [3]. The SGA is the weight below the 10th percentile of weight for the gestational age. Malaria accounts for 14 to 25% of LBW in sub-Saharan Africa [4–6]. LBW is a result of a short gestational period, IUGR or a combination of both processes, and contributes globally to high neonatal and infant mortality and morbidity [1, 3]. Particularly in sub-Saharan Africa (SSA), LBW neonates are nine times more likely to die in the 1st month of life than a normal-weight baby [7, 8]. Infants who are growth-restricted experience higher rates of fetal and infant death, birth asphyxia, hypothermia, hypoglycaemia, meconium aspiration, and long-term neurological impairment [9].

Cohort studies in malaria-endemic areas show an association between malaria infection at an earlier gestational age, i.e., in the first trimester, and adverse fetal growth, pregnancy outcome, duration of pregnancy, and placental weight at term [10, 11]. Walker et al. estimated that 65.2% (95% CI 60.9–70.0) of placental malaria infections occur at the end of the first trimester, and called for targeting this period for prevention [4]. Multiple measures to prevent and treat malaria and its complications during pregnancy are recommended. These preventive measures presently include the use of long-lasting insecticide-treated nets (LLINs), administration of intermittent preventive treatment in pregnancy with sulfadoxine–pyrimethamine (IPTp-SP), and rapid diagnosis and management of malaria cases [12]. The World Health Organization (WHO) currently recommends the use of artemisinin-based combination therapy (ACT) for the treatment of uncomplicated *P. falciparum* malaria in pregnant women in their second or third trimester. ACT is presently only recommended by WHO in the first trimester if quinine cannot be used or in case of severe malaria where the benefit outweighs the potential risk. Quinine is recommended for uncomplicated malaria in the first trimester of pregnancy [13]. However, quinine therapy has been documented to be associated with low adherence due to tolerability (occurrence of tinnitus, hearing impairment, dizziness, and postural hypotension) and need for multiple doses (3 times a day) for 7 days [12, 14].

Findings from preclinical studies have reported that artemisinins are embryotoxic and teratogenic in multiple animal species [15–18]. In settings where malaria is endemic and ACT is highly available in the market, it is likely that a woman will be inadvertently or intentionally exposed in the first trimester because, for example, women and health care providers may be unaware of a woman’s pregnancy status at the time of prescribing an anti-malarial [19–21]. Therefore, it is imperative to assess the safety of first trimester ACT exposures for a broad range of pregnancy outcomes. The assessment of safety of anti-malarial drug use during early pregnancy (ASAP) study was a multi-country prospective cohort study of pregnant women to evaluate whether ACT exposure in early pregnancy increases the risk of miscarriage, stillbirths, congenital malformations, and LBW when compared to current therapeutic options [22]. The risk of miscarriage, stillbirths and congenital anomalies has been reported elsewhere [23, 24]. This analysis aimed to evaluate the association between ACT exposure during the first trimester of pregnancy and LBW and SGA among the offspring of pregnant women.

## Methods

### Study design

ASAP was a prospective cohort study of pregnant women, conducted under a single multi-centre study protocol at three SSA sites as part of Malaria in Pregnancy Consortium activities as previously published [22]. The sites were located in Asembo-Siaya County, Kenya Nanoro, Burkina Faso; and, Manhiça District, Mozambique. In all three sites, malaria transmission is intense and *P. falciparum* is the main species. All three ASAP sites have a health and demographic surveillance system (HDSS) [25–28]. Within their defined communities, HDSS sites ensure recording of all vital status (births, deaths and migration) and other demographic events such as pregnancy by full enumeration of the population at least twice a year using community key informants [28]. Additional recruitment and data collection strategies were employed for the ASAP study to identify pregnancies, anti-malarial exposures, determine gestational age at the time of exposure to anti-malarials, monitor pregnancy outcomes, and systematically assess infant outcomes. The emphasis was placed on identifying first-trimester pregnancies by identifying and recruiting women as early as possible in pregnancy.

Pregnant women were identified through household visits, community key informants, and at antenatal care visits in a health facility within the HDSS catchment area. All identified pregnant women were invited to the antenatal care (ANC) clinics and assessed for eligibility. Following written consent, baseline information was collected and data entered into a pregnancy register. Electronic records from outpatient and inpatient visits were recorded through the HDSS platform and linked to the study records to identify possible exposure to ACT and other anti-malarials during the first trimester of the pregnancy. For this analysis, only singleton newborns with birth weight collected within the first 7 days of life are included. Multiple methods were used for the ascertainment of gestational age, including date of last menstrual period, fundal height, ultrasound, and Ballard Score as explained elsewhere [22]. Women were encouraged to deliver at the closest health facility where systems were in place to identify and link records. Also, deliveries occurring outside the health facility were actively identified by close monitoring lists of probable delivery and home-based visits or by notification from village informants and traditional birth attendants (TBAs) whereby a study staff team assessed cases delivered at home as soon as possible.

### Anti-malarial exposure group definition

The ascertainment of drug exposure included both prospective and retrospective self-reported medication usage and linkage to treatment records at local health facilities of drug prescribing and dispensing. The process of drug identification, self-reporting and record linkage with health facility data has been described elsewhere [22]. Two or more sources were required to confirm anti-malarial exposure; unconfirmed exposures were excluded. The treatments of interest were ACT or quinine during the first trimester of pregnancy, i.e., weeks 2 to 13 (inclusive) from the last menstrual period. Artemether–lumefantrine was the only ACT used in the three sites.

### Outcomes

The present analysis is restricted to birth weight, LBW and SGA among live births. LBW was defined as a birth weight of < 2500 g collected within the first 24 h of life [3]. Birth weights taken between 24 and 48 h and 48 to 168 h after delivery were corrected by a factor + 2% and + 4%, respectively, to obtain the estimated weight at birth [29–32]. SGA was defined as a corrected birth weight below the 10th percentile of weight for the gestational age using the INTERGROWTH reference curves [33, 34]. Following a suggestion by the reviewers, the analysis of prematurity was conducted as a supplemental analysis. Prematurity was defined as a live birth before 37 completed weeks of gestation [35].

### Statistical methods

Baseline characteristics were compared by site and by anti-malarial exposure to assess imbalances across sites and exposure groups. Frequencies were used for categorical variables and for numeric variables means and standard deviations. The prevalence of LBW and of SGA and the mean birth weight by exposure group in each site were computed. As measure of association, the mean difference (MD) was used for birth weight outcome and prevalence ratios (PR) for the prevalence of LBW and SGA. Two types of data synthesis of effect were performed. One is the site-level aggregated through the use of both fixed effects and random effects meta-analysis. Inverse variance based weights were used and for zero events study, 0.5 continuity correction was employed. To assess the heterogeneity between sites the I^2^ statistic was used [36].

The other data synthesis is based on patient-level data whereby linear (for MD) and log-binomial (for PR) regressions were used. All models included the site indicator as covariates. The age at recruitment, gravidity, marital status, and education level were included in the adjusted analysis. The log-binomial regressions had convergence failure and data spasticity issues. Thus, a Bayesian implementation of the log-binomial with posterior distributions approximated through Markov Chain Monte Carlo (MCMC) was performed in JAGS software [37–39]. Uninformative priors for the coefficients were set as normal distributions with 0 mean and 1000 variance, the MCMC were run with 3 chains of 100,000 iterations with 10,000 iterations as burn-in, and 50 as the thin steps. The convergence was assessed through the Gelman and Rubin’s diagnostic (Rhat < 1.2), review of the traceplots (assess the mixture of the simulations) and autocorrelation of the iterations [37, 38]. The significance level was set at 5%. Additional analyses and data preparation were performed on Stata v14 (StataCorp. 2015. Stata: Release 14. Statistical Software. College Station, TX, USA: StataCorp LP), R [40]. The INTERGROWTH-21st software was used to produce the percentiles of weights for gestation age [33].

### Ethical approval

The protocol was reviewed and approved by the Ethical Review Boards of the Kenya Medical Research Institute (KEMRI), US Centers for Disease Control and Prevention (CDC), National Bioethics Committee in Mozambique, Centre Muraz Institutional Ethics committee and National Ethics committee in Burkina Faso, Liverpool School of Tropical Medicine in the UK, and the Institutional Review Board of the University of Washington.

## Results

**Fig. 1:**
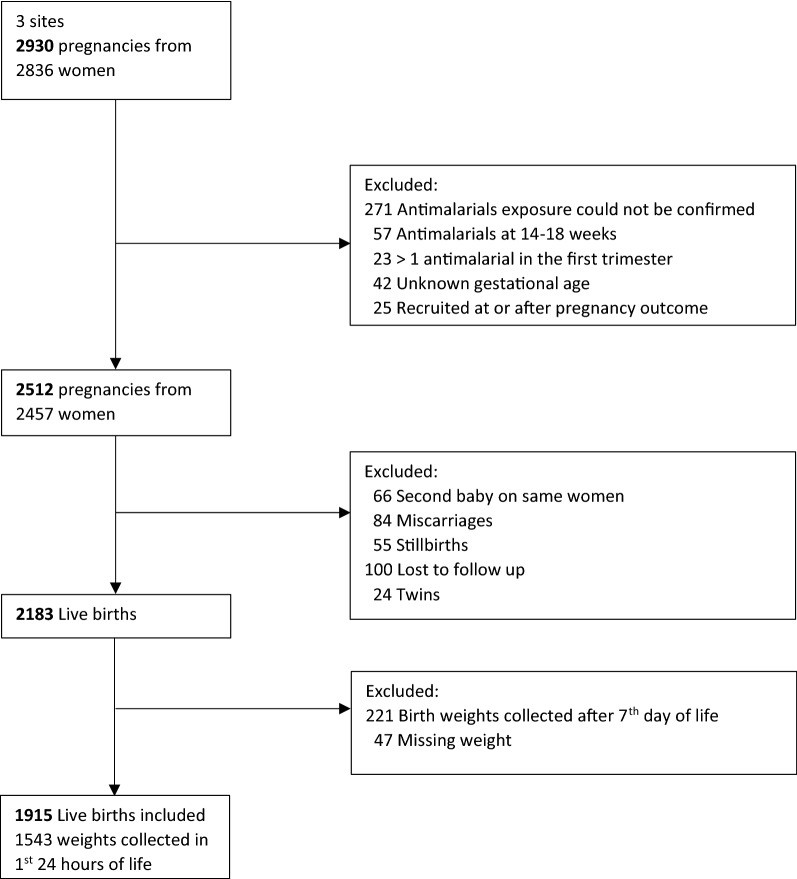
Flow chart of the recruited participants in ASAP cohort included in this analysis, 2015

A total of 2836 recruited women representing 2930 pregnancy outcomes and 1915 live births were included in the analysis (Fig. 1). Excluded from the analysis were records that lacked confirmation of anti-malarial exposure (271), birth weights collected past 7th day of life (221), those lost to follow-up (100), either miscarriages or stillbirths (139), either twins gestation or second pregnancy follow-up (90) and other reasons (194) (Fig. 1). Each site contributed similar numbers of subjects for analysis, 656, 669 and 590 live births from Burkina Faso, Mozambique and Kenya, respectively. Of the live births included in the analysis 80.6% (1543) had birth weight collected in the first day of life with across site values of 47.8% (282/590), 92.2% (605/656), and 98.1% (656/669) in Kenya, Burkina Faso and Mozambique, respectively. The final sample in terms of anti-malarial exposure during the first trimester of pregnancy included 1797 live births not exposed to an anti-malarial, 92 exposed to ACT and 26 exposed to quinine.

### Baseline characteristics

Demographic characteristics among participants are reported in Tables 1 and 2 per site and exposures, respectively. The average age at recruitment of the pregnant women in Burkina Faso was 27.1 years, which is almost 2 years older than in Kenya (25.7) and 3 years older than in Mozambique (24.2). There was 30.8% primigravidae in the quinine-exposed group compared to 25.0% among those who were ACT-exposed and 21.0% among the unexposed to anti-malarials group. Approximately 21.7% of deliveries were in-home deliveries in Kenya compared to 11.7% and 5.2% in Burkina Faso and Mozambique, respectively.

**Table 1:** Baseline characteristics of pregnancies included for low birth weight and small for gestational analysis per study site, ASAP cohort, 2015

Characteristic	Burkina Faso	Mozambique	Kenya
	N (%)	N (%)	N (%)
Total participants	656 (100)	669 (100)	590 (100)
Age at recruitment (years)			
Range	15.0–49.0	12.4–41.9	15.0–45.0
Mean (SD)	27.1 (6.64)	24.2 (6.23)	25.7 (6.48)
< 20	94 (14.3)	207 (30.9)	117 (19.8)
20–24	158 (24.1)	184 (27.5)	160 (27.1)
25–29	171 (26.1)	152 (22.7)	147 (24.9)
30+	233 (35.5)	126 (18.8)	166 (28.1)
Gravidity			
Primigravida	113 (17.2)	178 (26.6)	118 (20.0)
1–3 pregnancies	307 (46.8)	395 (59.0)	297 (50.3)
4 or more pregnancies	236 (36.0)	93 (13.9)	175 (29.7)
Missing	0 (0.0)	3 (0.4)	0 (0.0)
Marital status			
Single	9 (1.4)	240 (35.9)	125 (21.2)
Married or cohabiting	647 (98.6)	428 (64.0)	465 (78.8)
Missing	0 (0.0)	1 (0.1)	0 (0.0)
Education			
Primary not completed	0 (0.0)	96 (14.3)	268 (45.4)
Primary completed	656 (100.0)	330 (49.3)	276 (46.8)
Secondary completed	0 (0.0)	241 (36.0)	46 (7.8)
Missing	0 (0.0)	2 (0.3)	0 (0.0)
HIV status			
Negative	633 (96.5)	442 (66.1)	445 (75.4)
Positive	4 (0.6)	164 (24.5)	125 (21.2)
Missing	19 (2.9)	63 (9.4)	20 (3.4)
Gestational age at recruitment (weeks)			
Mean (SD)	24.0 (6.15)	21.2 (5.65)	17.9 (10.20)
Median (IQR)	24.1 (19.6–28.7)	21.0 (17.0–25.0)	15.9 (9.1–26.0)
Place of delivery			
Health facility	579 (88.3)	611 (91.3)	424 (71.9)
Home	77 (11.7)	35 (5.2)	128 (21.7)
Other	0 (0.0)	23 (3.4)	38 (6.4)

**Table 2:** Baseline characteristics by exposure level (no exposure, exposed to ACT, exposed to quinine), ASAP cohort, 2015

Characteristic	No anti-malarial use in first trimester	Confirmed ACT use in first trimester	Confirmed quinine use in first trimester	All pregnancies
	N (%)	N (%)	N (%)	N (%)
Total	1797 (100)	92 (100)	26 (100)	1915 (100)
Country				
Burkina Faso	603 (33.6)	31 (33.7)	22 (84.6)	656 (34.3)
Mozambique	643 (35.8)	22 (23.9)	4 (15.4)	669 (34.9)
Kenya	551 (30.7)	39 (42.4)	0 (0.0)	590 (30.8)
Age at recruitment (years)				
Range	12.4–49.0	15.0–45.0	17.0–36.0	12.4–49.0
Mean (SD)	25.7 (6.55)	25.6 (6.63)	24.7 (6.04)	25.6 (6.54)
< 20	394 (21.9)	17 (18.5)	7 (26.9)	418 (21.8)
20–24	465 (25.9)	30 (32.6)	7 (26.9)	502 (26.2)
25–29	444 (24.7)	19 (20.7)	7 (26.9)	470 (24.5)
30+	494 (27.5)	26 (28.3)	5 (19.2)	525 (27.4)
Gravidity				
Primigravida	378 (21.0)	23 (25.0)	8 (30.8)	409 (21.4)
1–3 pregnancies	943 (52.5)	45 (48.9)	11 (42.3)	999 (52.2)
4 or more pregnancies	473 (26.3)	24 (26.1)	7 (26.9)	504 (26.3)
Missing	3 (0.2)	0 (0.0)	0 (0.0)	3 (0.2)
Marital status				
Single	354 (19.7)	20 (21.7)	0 (0.0)	374 (19.5)
Married or cohabiting	1442 (80.2)	72 (78.3)	26 (100.0)	1540 (80.4)
Missing	1 (0.1)	0 (0.0)	0 (0.0)	1 (0.1)
Education				
Primary not completed	348 (19.4)	16 (17.4)	0 (0.0)	364 (19.0)
Primary completed	1176 (65.4)	62 (67.4)	24 (92.3)	1262 (65.9)
Secondary completed	271 (15.1)	14 (15.2)	2 (7.7)	287 (15.0)
Missing	2 (0.1)	0 (0.0)	0 (0.0)	2 (0.1)
HIV status				
Negative	1427 (79.4)	70 (76.1)	23 (88.5)	1520 (79.4)
Positive	279 (15.5)	13 (14.1)	1 (3.8)	293 (15.3)
Missing	91 (5.1)	9 (9.8)	2 (7.7)	102 (5.3)
Gestational age at recruitment (weeks)				
Mean (SD)	21.3 (7.39)	18.8 (8.72)	16.8 (5.56)	21.2 (7.88)
Gestational age at delivery (weeks)				
Mean (SD)	38.9 (1.80)	39.0 (1.77)	38.8 (1.65)	38.9 (1.79)

*SD* standard deviation

### Birth weight outcome

**Fig. 2:**
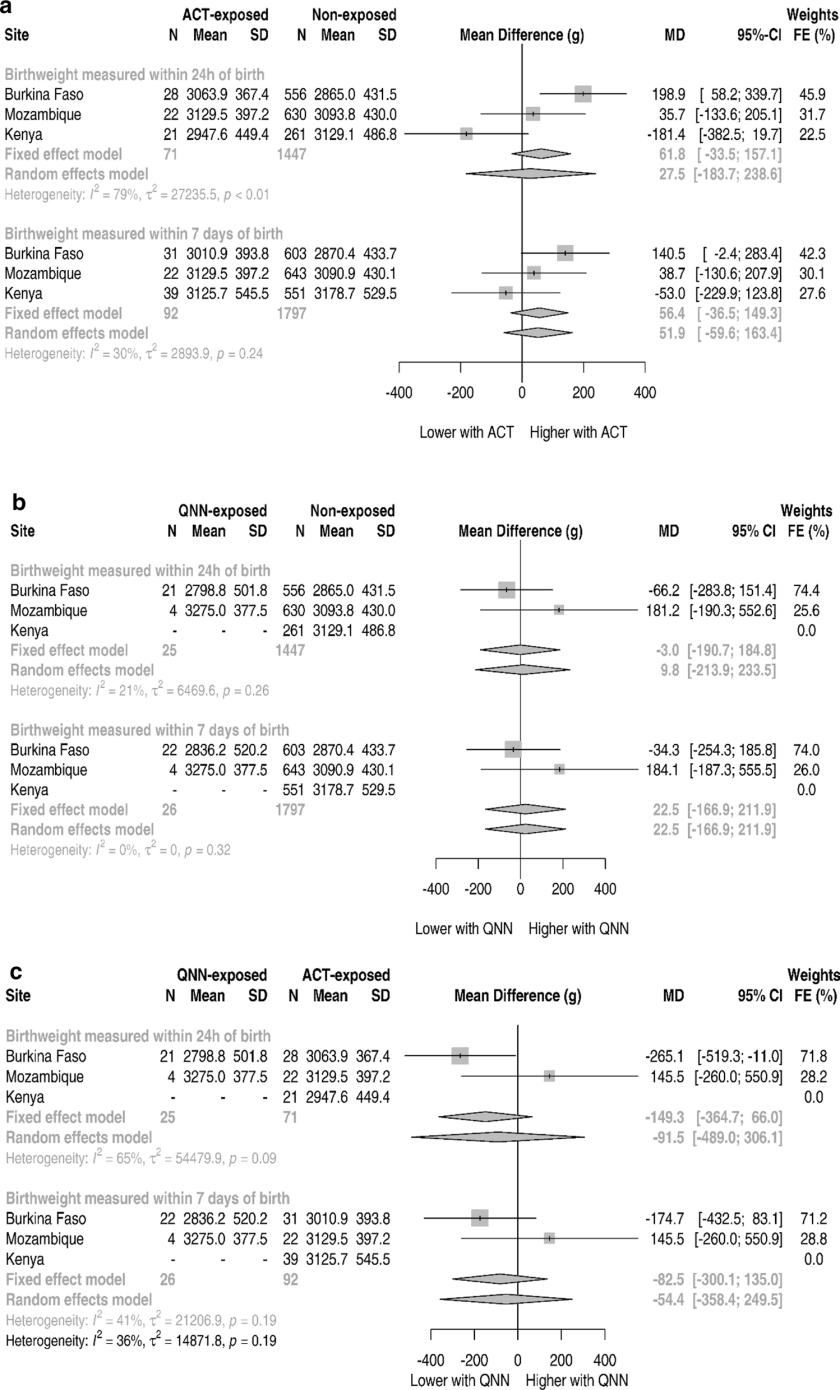
Weight at birth mean difference in grams for different sites and random-effects pooled association, ASAP study

Newborns at Burkina Faso’s site had on average a weight of 2875.9 g, compared to Mozambique’s and Kenya’s site with 3093.3 g and 3175.2 g, respectively. Figure 2 and Additional file 1: Table S4 show the aggregated data synthesis of mean differences of weights. For birth weight collected in the 1st week of life, the pooled fixed effects mean-difference between ACT and non-exposed group was found to be 56.4 g (95% CI − 36.5 to 149.3, p-value 0.234) whereas for random-effects it was 51.9 g (95% CI − 59.6 to 163.4, p-value 0.362), both values are not significantly different from the null. These results did not appreciably differ from those collected only within the first 24 h of life (Fig. 2a and Additional file 1: Table S4). For the comparison between quinine and non-exposed (Fig. 2b and Additional file 1: Table S5), Kenya could not be included due to the lack of quinine exposure, the contribution of the remaining sites gave both pooled fixed and random effects of a non-significant association of 22.5 g (95% CI − 166.9 to 211.9, p-value 0.816) for birth weights collected in the first week. When examining just the birth weights collected within the first day of life the differences reduce in magnitude to just − 3.0 g and 9.8 g for fixed-effects and random-effects, respectively. Figure 2c and Additional file 1: Table S6 show the comparison between quinine and ACT exposures. All associations are in the direction of higher birth weights for ACT although not statistically significant with the fixed-effects of − 82.5 g (95% CI − 300.1 to 135.0, p-value 0.457) for birth weights collected in the first week. Except for quinine versus non-exposed comparison, the heterogeneity of all these associations is between moderate to high (I^2^ above 25%).

Table 3 shows the mean difference for individual-level analysis. For the quinine versus ACT comparison using the birth weights collected within the first week of life the unadjusted mean difference was 108.0 g (95% CI − 341.2 to 125.1, p-value − 0.361) in favour of the ACT group. This MD increased slightly in favor of the ACT with a value of − 167.7 g (95% CI − 391.6 to 56.1, p-value 0.140) when adjusted for other covariates.

**Table 3:** Adjusted associations for mean weight at birth

	Mean difference in g (95% CI)			
	Unadjusted^a^	p-value	Adjusted^b^	p-value
Birth weights measured within 24 h of birth (N)	1543		1533	
No exposure	0 (reference)		0 (reference)	
Artemisinin	37.8 (− 67.7; 143.3)	0.482	29.6 (− 62.0; 121.3)	0.526
Quinine	− 32.1 (− 207.9; 143.8)	0.721	− 29.0 (− 181.6; 123.6)	0.709
Birth weights measured within 7 days of birth (N)	1915		1905	
No exposure	0 (reference)		0 (reference)	
Artemisinin	34.9 (− 62.7; 132.5)	0.483	40.0 (− 47.8; 127.8)	0.371
Quinine	− 4.2 (− 185.7; 177.2)	0.964	− 3.6 (− 166.8; 159.6)	0.965
*Quinine vs artemisinin (N)*	118		118	
Artemisinin	0 (reference)		0 (reference)	
Quinine	− 108.0 (− 341.2; 125.1)	0.361	− 167.7 (− 391.6; 56.1)	0.140

^a^Unadjusted regression includes dummy indicators for site

^b^Adjusted for site, age at recruitment, gravidity, marital status and education level

### LBW outcome

**Fig. 3:**
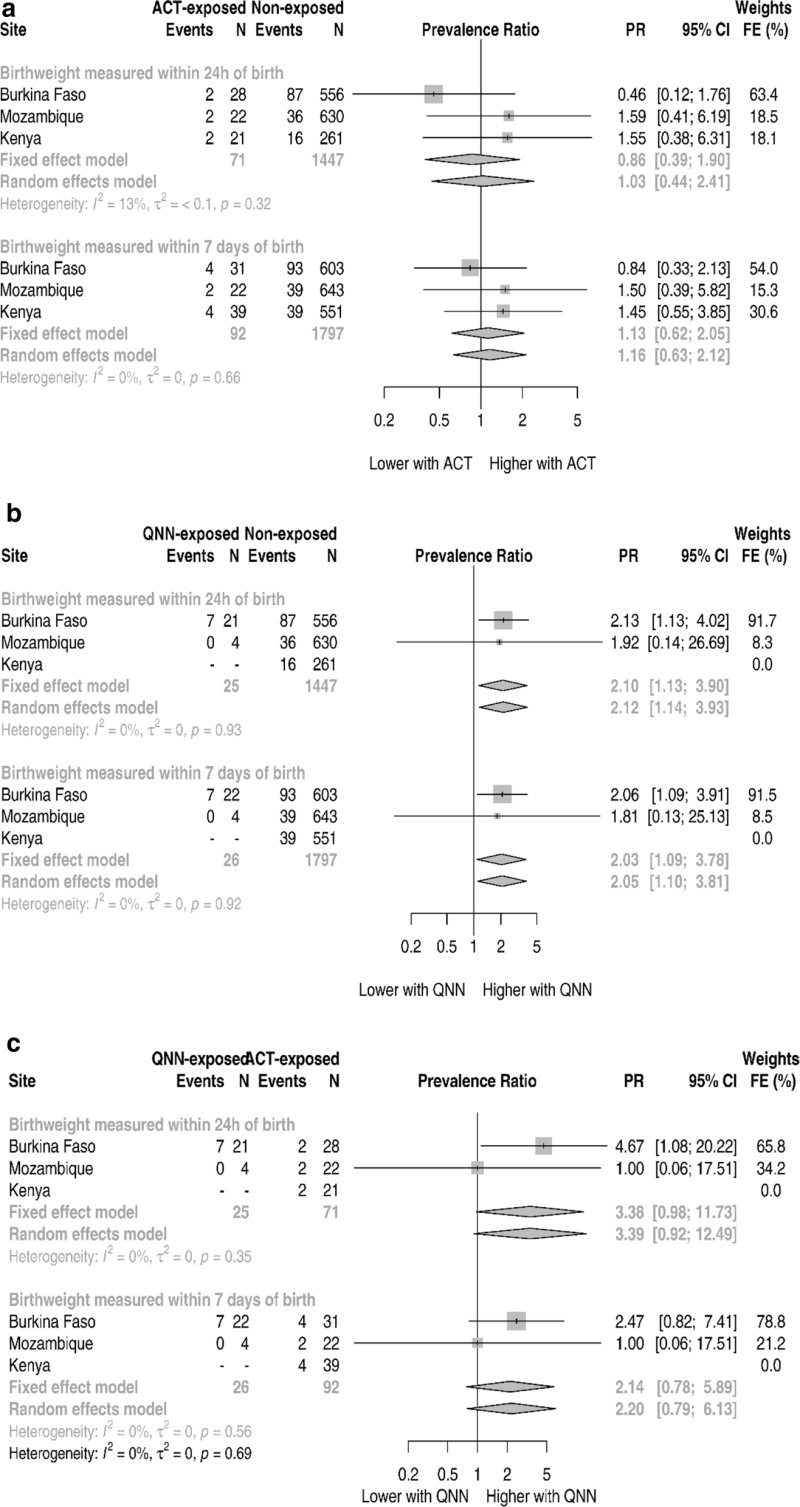
ASAP site specific and pooled low birth weight prevalence ratios

The prevalence of LBW was 15.9% (104/656), 6.1% (41/669) and 7.2% (43/590) in Burkina Faso, Mozambique and Kenya, respectively. Figure 3 shows the pooling of the site-aggregated data. Relative to non-exposed, newborns exposed to ACT (Fig. 3a and Additional file 1: Table S7) with weight collected in the first week of life had 1.13 (95% CI 0.62–2.05, p-value 0.700) times higher prevalence of LBW through the fixed-effects estimate. A similar magnitude was found through the random-effects estimate. In Kenya, there was no quinine exposure. Relative to non-exposed newborns, quinine exposed had 2.03 (95% CI 1.09–3.78, p-value 0.027) times higher prevalence of LBW through the fixed-effects estimate (Fig. 3b and Additional file 1: Table S8). A similar association was found through the random-effects. Compared to ACT, the quinine-exposed newborns had a non-significant 2.14 (95% CI 0.78–5.89, p-value 0.142) times higher prevalence of LBW through the fixed-effects analysis. Similar results were obtained on the random-effects analysis. Furthermore, this association becomes stronger, although marginally significant when restricting to weights collected within the first 24 h of birth (Fig. 3c and Additional file 1: Table S9). A small heterogeneity was observed for LBW aggregated analysis with all I^2^ below 15%.

Table 4 shows the results of the individual-level analysis for the LBW outcome. There were no differences in the prevalence of LBW for pregnancies treated with quinine compared to those treated with an ACT in the first trimester (unadjusted PR 2.03, 95% CI 0.73–5.86, p-value 0.172). The association reduced to 1.70 (95% CI 0.60–5.17, p-value 0.321) with adjustment for covariates.

**Table 4:** Adjusted associations for low birth weight and small for gestational age

	Prevalence-ratio (95% CI^c^)			
	Unadjusted^a^	p-value	Adjusted^b^	p-value
LBW				
Birth weights measured within 24 h of birth (N)	1543		1533	
No exposure	1 (reference)		1 (reference)	
Artemisinin	0.80 (0.31; 1.62)	0.575	0.91 (0.36; 1.81)	0.809
Quinine	1.95 (0.87; 3.42)	0.099	2.17 (1.00; 3.65)	0.049
Birth weights measured within 7 days of birth (N)	1915		1905	
No exposure	1 (reference)		1 (reference)	
Artemisinin	1.08 (0.55; 1.85)	0.806	1.19 (0.61; 2.00)	0.579
Quinine	1.87 (0.85; 3.27)	0.109	2.13 (0.98; 3.59)	0.055
*Quinine vs artemisinin*				
Artemisinin	1 (reference)		1 (reference)	
Quinine with no Kenya data (N = 79)	2.03 (0.73; 5.86)	0.172	1.70 (0.60; 5.17)	0.321
Quinine with Kenya data (N = 118)	3.18 (1.02; 11.56)	0.047	1.93 (0.72; 5.68)	0.188
SGA				
Birth weights measured within 24 h of birth (N)	1520		1514	
No exposure	1 (reference)		1 (reference)	
Artemisinin	0.76 (0.37; 1.31)	0.363	0.83 (0.41; 1.42)	0.516
Quinine	1.31 (0.54; 2.43)	0.495	1.19 (0.50; 2.17)	0.660
Birth weights measured within 7 days of birth (N)	1888		1882	
No exposure	1 (reference)		1 (reference)	
Artemisinin	0.81 (0.43; 1.31)	0.417	0.78 (0.43; 1.25)	0.337
Quinine	1.29 (0.55; 2.40)	0.514	1.11 (0.47; 2.02)	0.789
*Quinine vs artemisinin*				
Artemisinin confirmed	1 (reference)		1 (reference)	
Quinine confirmed with no Kenya data (N = 78)	13.75 (2.16; 409.72)	0.003	13.11 (1.82; 441.93)	0.004
Quinine confirmed with Kenya data (N = 116)	14.44 (2.18; 400.29)	0.004	12.04 (1.84; 330.23)	0.008

*LBW* low birth weight, *SGA* small for gestational age

^a^Unadjusted regression includes dummy indicators for site

^b^Adjusted for site, age at recruitment, gravidity, marital status and education level

^c^95%CI—95% credible interval based on § posterior distribution replication

### Small for gestational age and prematurity

**Fig. 4:**
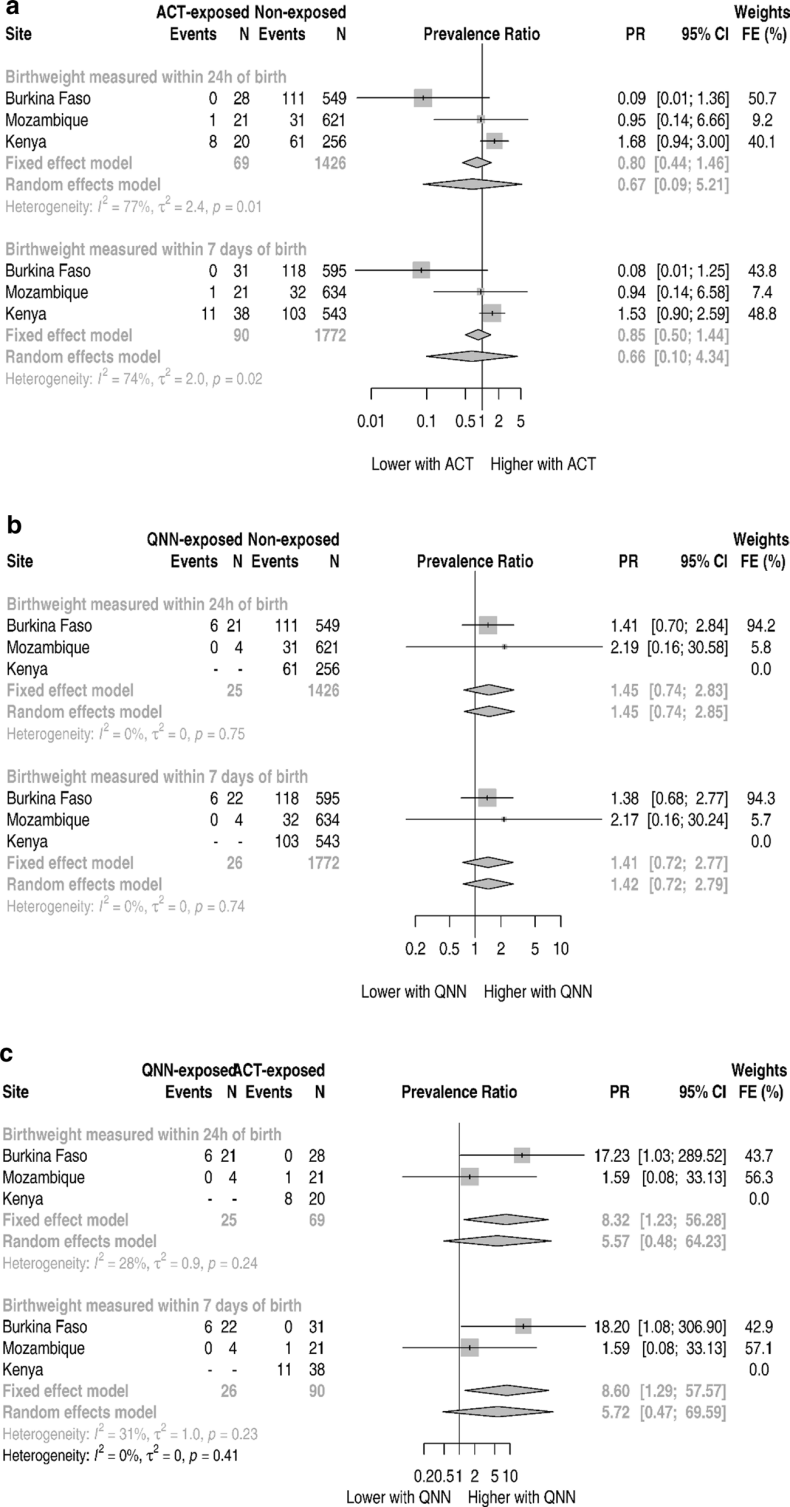
ASAP site specific and pooled small for gestation age prevalence ratios

The prevalence of SGA was 19.1% (124/648), 5.0% (33/659) and 19.6% (114/581), respectively in Burkina Faso, Mozambique and Kenya. Figure 4 and Additional file 1: Tables S10–S12 show the forest plots for the site aggregated analysis for SGA prevalence ratio. Across the three sites, quinine-exposed newborns had a 41% (1.41, 95% CI 0.72–2.77, p-value 0.322) relatively higher prevalence of SGA than non-exposed under the fixed-effects pooling (Fig. 4b and Additional file 1: Table S11). The magnitude of the association increased to 8.60 (95% CI 1.29–57.57) for the quinine versus ACT comparison (Fig. 4c). Pregnancies treated with ACT during the first trimester were not associated with an increased prevalence of SGA compared with pregnancies not treated with an anti-malarial (PR 0.85, 95% CI 0.50–1.44) (Fig. 4a).

Table 4 shows the individual level based analysis for the SGA prevalence. On the unadjusted analysis, quinine-exposed compared to ACT-exposed newborns had 13.75 times higher prevalence of SGA with a wide confidence interval (2.16–409.72) not including the null, with similar results under the adjusted analysis.

**Fig. 5:**
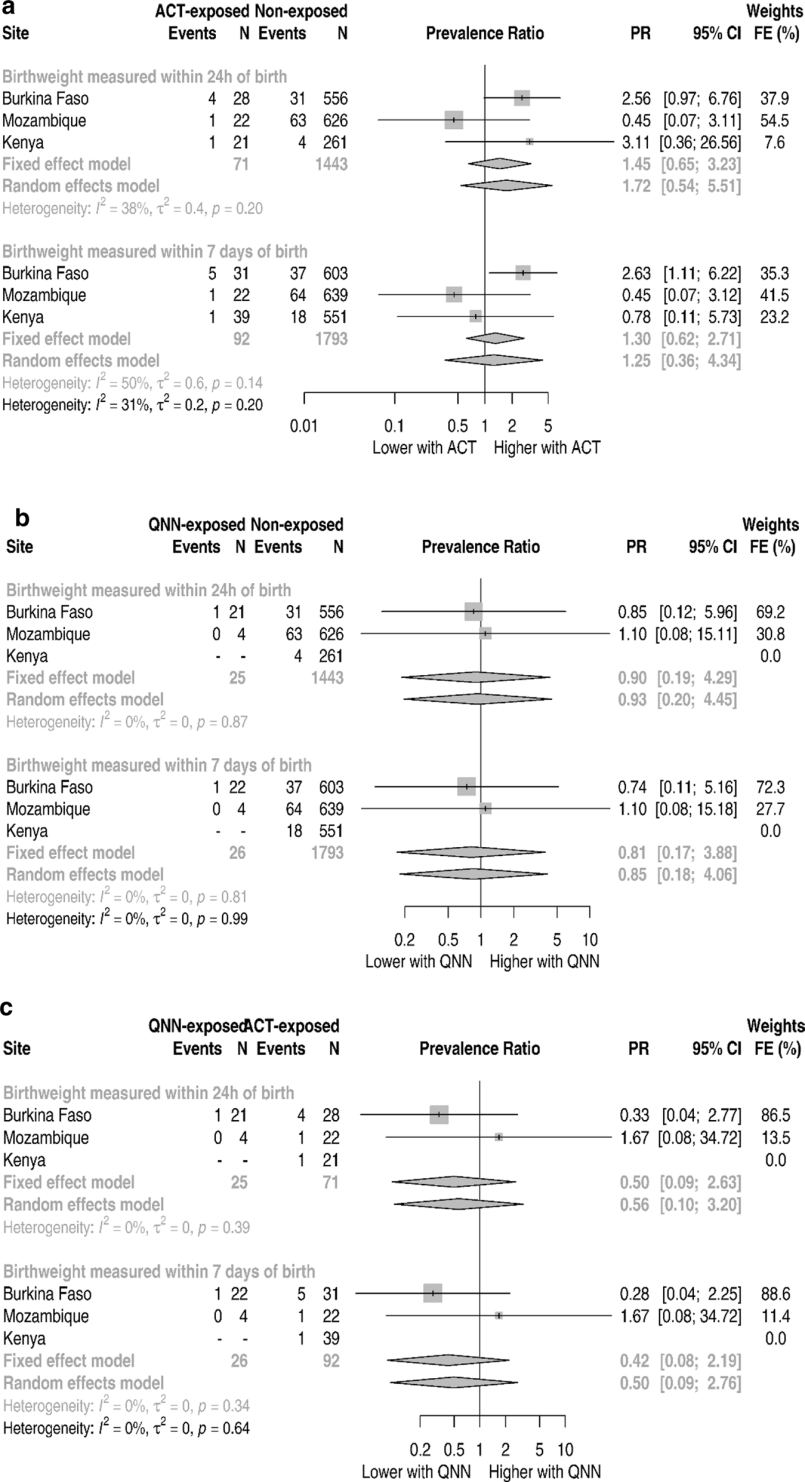
ASAP site specific and pooled prematurity prevalence ratios

The prevalence of prematurity was 6.6% (43/656), 9.8% (65/665) and 3.2% (19/590) respectively in Burkina Faso, Mozambique and Kenya. Figure 5 and Additional file 1: Tables S13–S15 show the forest plots for the site aggregated analysis for prematurity prevalence ratio. Across the all sites, under the fixed-effects pooling none of the associations between either ACT- or quinine-exposed newborns, and prematurity reached statistical significance (Fig. 5b, c and Additional file 1: Tables S14 and S15). The individual level based analysis did not meaningfully change the results (Additional file 1: Table S16).

## Discussion

No evidence was found of an increased risk of LBW, SGA or prematurity among pregnancies with a confirmed exposure to ACT for malaria treatment during the first trimester of pregnancy is not associated with an increased risk of LBW, SGA and prematurity compared to newborns unexposed to anti-malarials. The findings on ACT use in early pregnancy and fetal growth add reassurance to the previously documented safety profile of ACT used during early pregnancy in many SSA countries [19, 41–43]. A meta-analysis that incorporated studies in SSA and Asia provided further evidence on the safety of ACT and the risk of miscarriages, stillbirth and congenital anomalies [24].

While infrequently used in this study, quinine exposure in the first trimester of pregnancy was associated with a twice higher prevalence of LBW when compared to the unexposed to anti-malarials group or ACT-exposed. This finding should be interpreted with caution since it may be due to small numbers in the sample or to inadequately treated malaria resulting from the known drawbacks with oral quinine related to poor tolerability characterized by tinnitus, hearing impairment, dizziness, and postural hypotension and need for multiple doses (3 times a day) for 7 days [14, 44], creating a channelling bias [45]. Furthermore, Mosha et al. reported a trend towards a protective but non-significant association on LBW of quinine exposure in the first trimester of gestation [41].

There was no evidence of an increased prevalence of SGA with exposure to ACT. This finding is consistent with Manyando et al. who reported a similar prevalence of SGA among newborns exposed to ACT compared to exposed to sulfadoxine–pyrimethamine (9.0 vs 7.7%) in a cohort in Zambia and using a more restrictive definition of SGA (weight for gestational age below the fifth percentile). Also, there was no evidence of an increased prevalence of pre-term with exposure to ACT. However, quinine is possibly associated with a higher prevalence of SGA compared to those unexposed to anti-malarials or ACT in the first trimester of gestation. Previous literature comparing these risks is limited; this is the first study to investigate this association using the recently available world reference growth curves during gestation [19, 33].

Although malaria in pregnancy is common in SSA, it can be easily confused with many other febrile diseases that may occur during pregnancy. It has been shown in Mozambique that only 27% of pregnant women presenting with fever had parasitaemia detected [46]. Moreover, there might be a high self-perceived risk of malaria among pregnant women in settings such as the ones where the study was conducted. All this may lead to the treatment of unconfirmed malaria with ACT given its higher availability [47]. Particularly, in pregnancy when early weeks of gestation are not disclosed due to cultural reasons or its unawareness, accidental anti-malarial exposure may occur more frequently than some studies have counted because only women with documented malaria are included [19, 41]. This calls for strengthening of pharmacovigilance systems in these settings.

The prevalence of SGA found in this study varied between 5.0% among infants born in Mozambique and 19.6% among infants born in Kenya. These estimates are consistent with recent estimates of SGA based on 22 birth cohort studies from the SGA-Preterm Birth working group [48]. However, a higher rate of SGA associated with *P. falciparum* infection was reported among pregnant women from the Thailand Myanmar border [49]. In this study, more than half of the women had their first antenatal visit during the second trimester of the pregnancy. This finding is very similar to DHS reports [50, 51]. However, Asembo is an exception given that at least a third of the recruited women had the first antenatal visit in the first trimester. Starting antenatal care early during pregnancy increases the likelihood of initiating IPTp-SP and bed nets in accordance with guidelines and thereby reducing the occurrence of malaria and need for use of quinine and ACT. HDSS procedures mandate frequent visits to a household. This increases the likelihood of detecting early pregnancies and facilitates follow-up of pregnancy outcomes. Nevertheless, cultural barriers [19] still pose challenges to the field workers to identify not yet visible pregnancies as almost half of the pregnancies were detected during the second semester.

## Limitations

This study included only pregnancies ending as singleton live birth and with birth weight collected within 7 days of life in the analysis. This could lead to some bias because SGA is associated with a lower probability of survival. Thus, the inclusion in the data analysis is conditioning on the outcome. However, it is not expected to be an important source of bias because the neonatal mortality rate in these sites is small (fewer than 30 per 1000 live-births). Compared to other study sites, Asembo (Kenya) had a higher proportion of missing information of weight at birth variable, representing 12% of all pregnancies recruited (Fig. 1). The vast majority of these missing birth weights are among newborns born at home, at which evaluation on the birthday was not possible. An imputation technique was used to mitigate this problem and the results did not materially change from the non-imputed scenario. However, the used imputation assumes that all newborns in one site are similar regardless of potential unmeasured biological differences (gender and mother anthropometrics). This may have contributed to lower prevalence of LBW because newborns who died due to conditions linked to LBW did not get their weights recorded and thus they do not contribute on the imputation. However, it is not expected to have resulted in changing the overall direction of the associations. The ascertainment of the gestational age and weight at birth in the context of this study may have introduced some non-differential misclassification. While gestational age was assessed via multiple methods, as available and appropriate, including date of last menstrual period, Ballard Score, fundal height and ultrasound, there is no reason to believe that the accuracy of gestational age estimates varied by exposure status. Interviewers and study nurses were unaware of exposure status [22]. Similarly, weight at birth was recorded without knowledge of exposure status.

Regarding birth weight, some newborns who were delivered in home settings had their weight assessed post-24 h of birth. If such non-differential misclassification occurred, it would have biased the estimates toward the null [19]. Nevertheless, the direction of the associations is the same as it was for the LBW. The sample size for quinine exposure is small and no exposures to quinine in Kenya were found. In addition, the main ACT used artemether–lumefantrine. Thus, one cannot necessarily generalize these results to other ACT medicines. Furthermore, the data on the malaria episode being treated was limited and there could be a risk of confounding if there were differences in the type of underlying infections being treated by quinine and ACT. Also, the models could not be adjusted for HIV because of small counts for HIV positives.

## Conclusions

No evidence was found of an increased risk of LBW, SGA or prematurity among infants born to women with confirmed first-trimester exposure to an ACT. The findings add support for the use of ACT for uncomplicated *P. falciparum* malaria during the first trimester of pregnancy. The existence of the HDSS platform greatly facilitated active safety surveillance of anti-malarials used during pregnancy.

## Supplementary information

**Additional file 1.** Additional Figures S1–S3, Tables S1–S16.

## Data Availability

The datasets generated during and/or analysed during the current study are available from the corresponding author on reasonable request.

## Abbreviations

ACT: Artemisinin-based combination therapy
ASAP: Assessment of safety of antimalarial drug use during early pregnancy
HDSS: Health and Demographic Surveillance System
INDEPTH: International Network of Demographic Evaluation of Populations and their Health
IPD: Individual Participant Data
IPTp: Intermittent preventive treatment in pregnancy
IUGR: Intrauterine growth restriction
LBW: Low birth weight
LLITN: Long-lasting insecticide-treated nets
QNN: Quinine
SSA: Sub-Saharan Africa
SP: Sulfadoxine–pyrimethamine
WHO: World Health Organization
