# Supplementary material for: First trimester use of artemisinin-based combination therapy and the risk of low birth weight and small for gestational age
Source: Malar J. 2020 Apr 8;19:144. doi: 10.1186/s12936-020-03210-y (PMC7140480; doi:10.1186/s12936-020-03210-y)
Supplement: Supplementary file 1 — Additional file 1. Additional Figures S1–S3, Tables S1–S16. [file 12936_2020_3210_MOESM1_ESM.docx]

Figure S1 – Cumulative unadjusted proportions of birth weights per antimalarial exposure across ASAP sites.


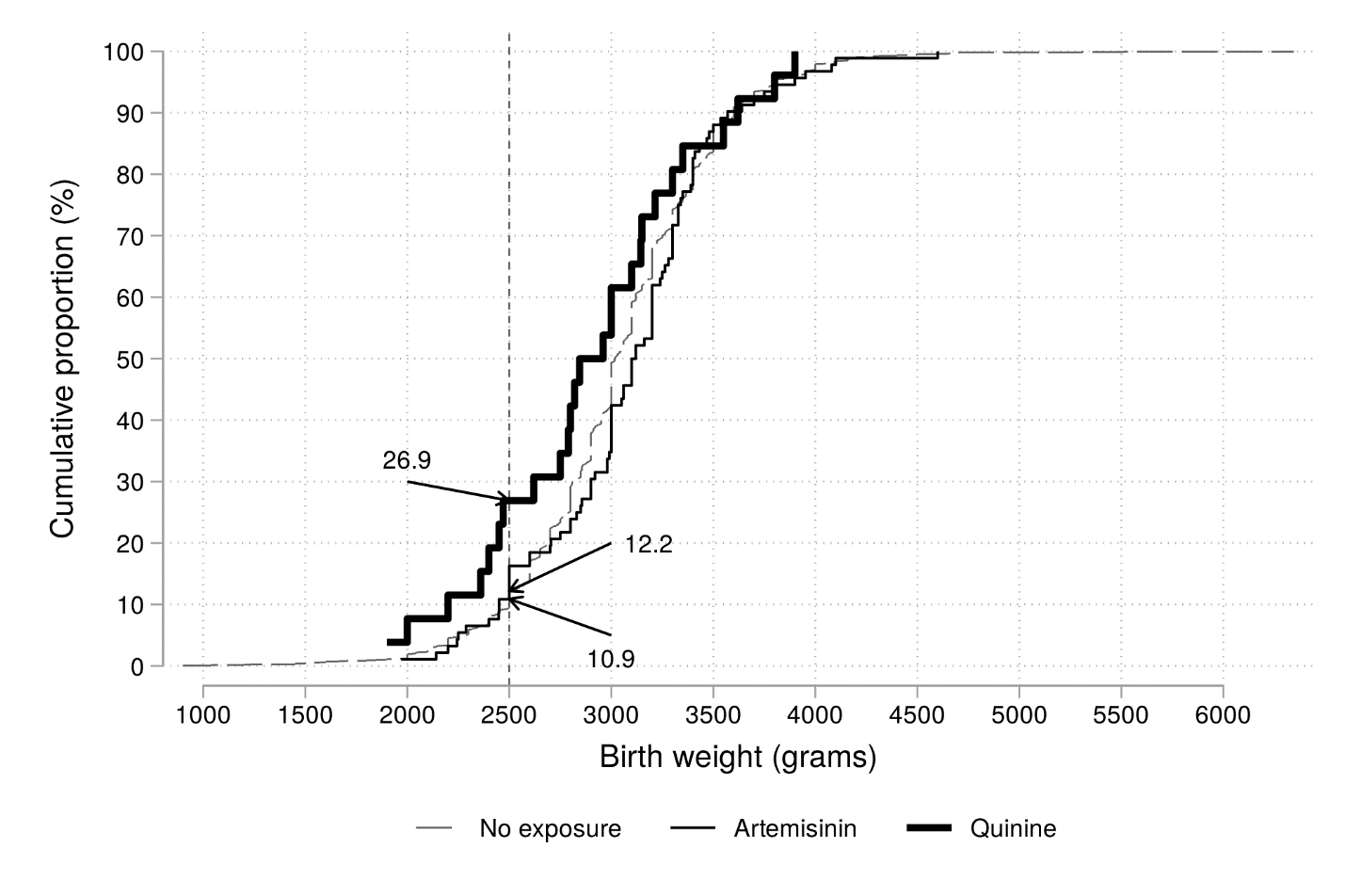


Note: Corrected to day 0 birth weights included. The vertical line indicates the threshold for low birth weight definition.

Figure S2 – Cumulative unadjusted proportions of percentiles of birth weight for gestational age per antimalarial exposure across all ASAP sites


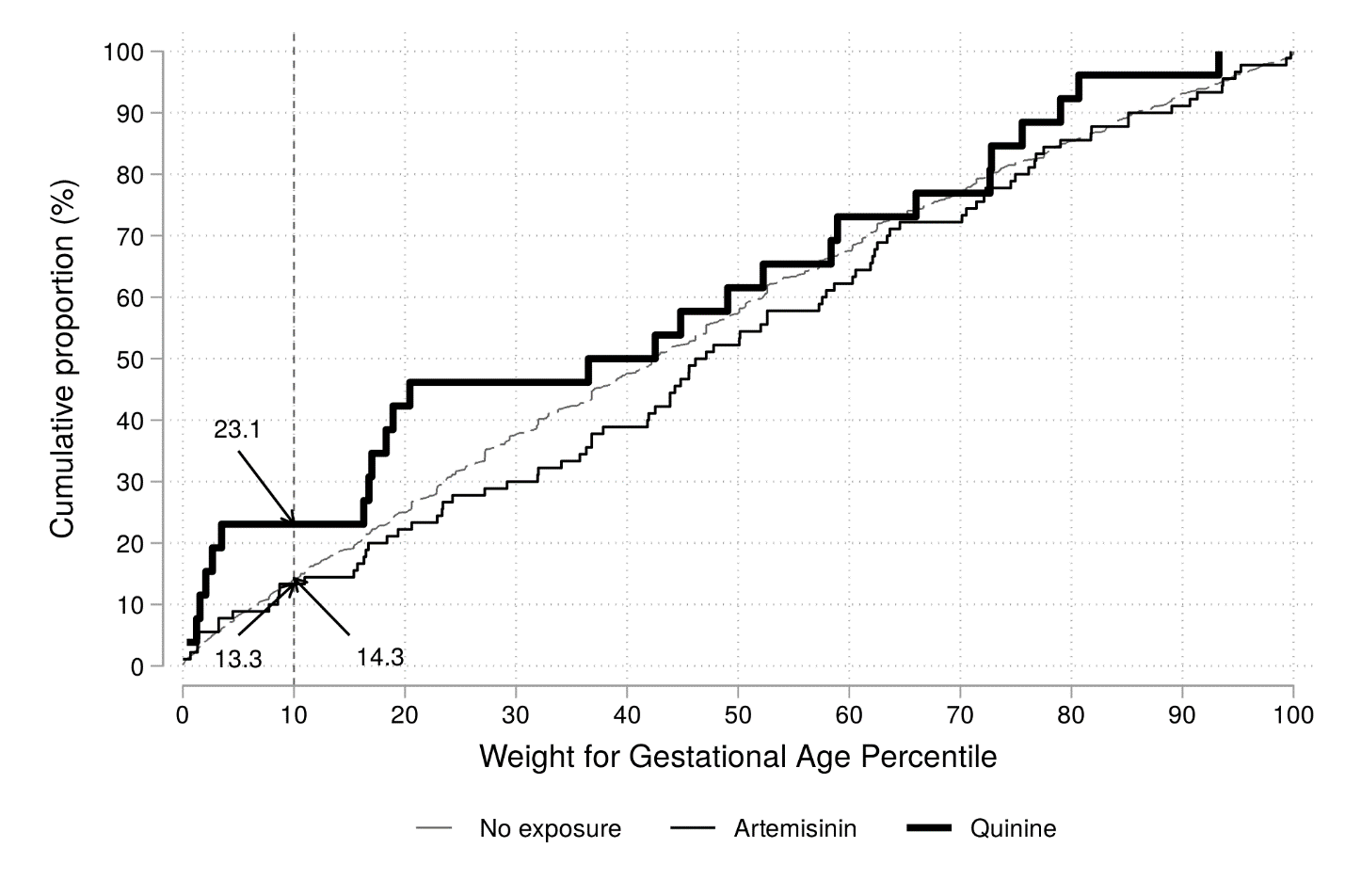


Note: Corrected to day 0 birth weights included. The vertical line indicates the threshold for small for gestational age. International reference curves are used to compute the percentiles[1].

Figure S3 – Cumulative unadjusted proportions of gestational age (in weeks) per antimalarial exposure across all ASAP sites


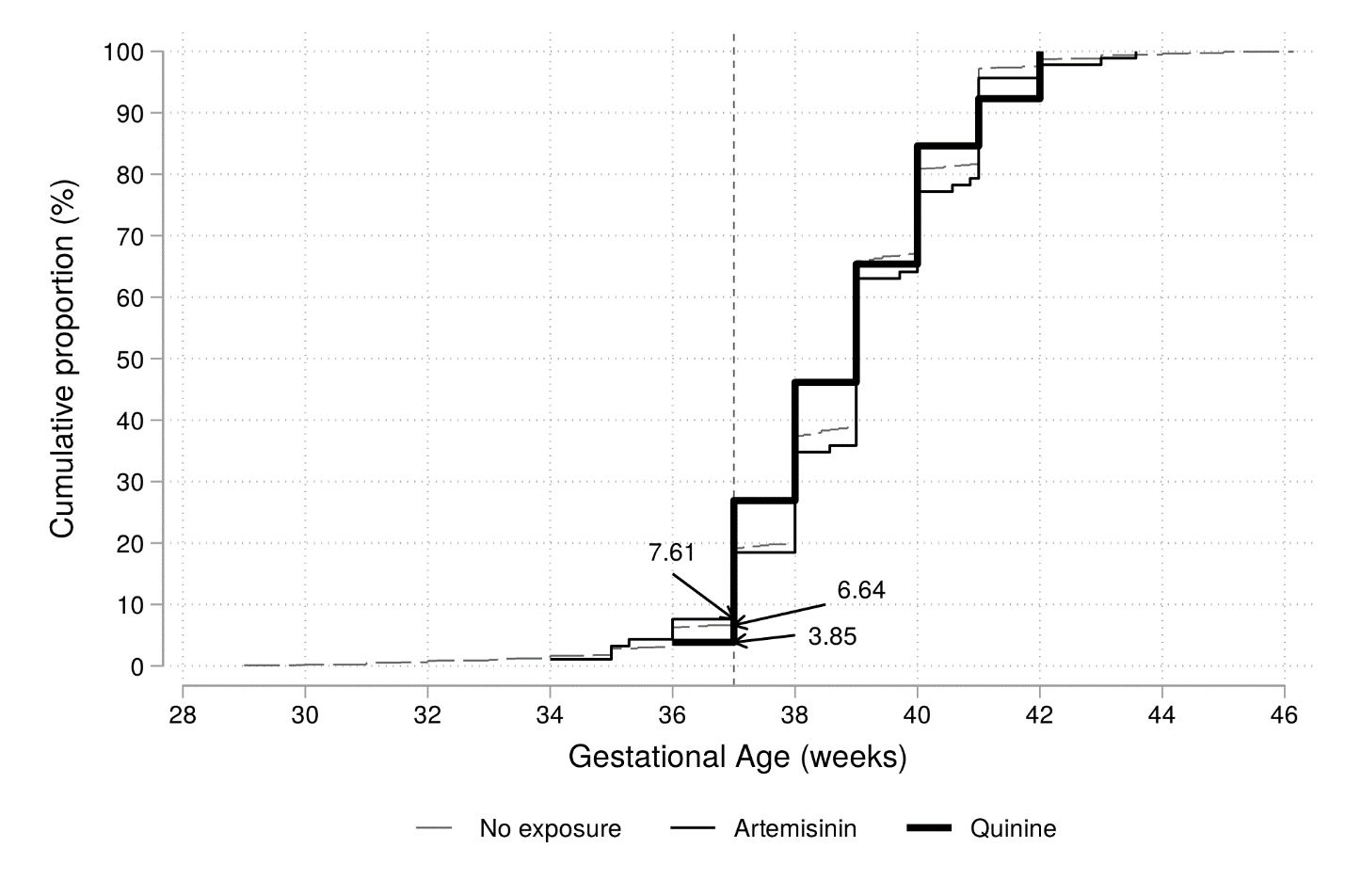


Table S1 - Mean birthweight in grams, by exposure status and study site, ASAP cohort

|  | **Burkina Faso** | | **Mozambique** | | **Kenya** | |
| --- | --- | --- | --- | --- | --- | --- |
|  | **N** | **mean (95%CI)** | **N** | **mean (95%CI)** | **N** | **mean (95%CI)** |
| **Total weights collected on day of birth** | **605** |  | **656** |  | **282** |  |
| **Weights collected on day of birth (grams)** |  |  |  |  |  |  |
| No exposure | 556 | 2865.0 (2829.1 - 2900.9) | 630 | 3093.8 (3060.2 - 3127.5) | 261 | 3129.1 (3069.7 - 3188.4) |
| Artemisinin | 28 | 3063.9 (2927.6 - 3200.3) | 22 | 3129.5 (2963.3 - 3295.8) | 21 | 2947.6 (2754.6 - 3140.7) |
| Quinine | 21 | 2798.8 (2583.7 - 3013.9) | 4 | 3275.0 (2904.4 - 3645.6) | 0 | - |
|  |  |  |  |  |  |  |
| **Total weights corrected to first day of life** | **656** |  | **669** |  | **590** |  |
| **Weights corrected to first day of life (grams)** |  |  |  |  |  |  |
| No exposure | 603 | 2868.0 (2833.4 - 2902.6) | 643 | 3090.5 (3057.2 - 3123.7) | 551 | 3178.7 (3134.4 - 3223.0) |
| Artemisinin | 31 | 3012.9 (2875.1 - 3150.8) | 22 | 3129.5 (2963.3 - 3295.8) | 39 | 3125.7 (2954.1 - 3297.3) |
| Quinine | 22 | 2836.2 (2618.4 - 3053.9) | 4 | 3275.0 (2904.4 - 3645.6) | 0 | - |
|  |  |  |  |  |  |  |

Table S2 - LBW prevalence and prevalence rate ratio, per exposure status and study site, ASAP cohort

|  | **Burkina Faso** | | **Mozambique** | | **Kenya** | |
| --- | --- | --- | --- | --- | --- | --- |
|  | **N (n)** | **Prevalence (95%CI)*** | **N (n)** | **Prevalence (95%CI)*** | **N (n)** | **Prevalence (95%CI)*** |
| **Total weights collected on day of birth** | **605 (96)** |  | **656 (38)** |  | **282 (18)** |  |
| **Prevalence of LBW using weights collected on day of birth** | |  |  |  |  |  |
| No exposure | 556 (87) | 15.6 (12.7 - 18.9) | 630 (36) | 5.7 (4.0 - 7.8) | 261 (16) | 6.1 (3.5 - 9.8) |
| Artemisinin | 28 (2) | 7.1 (1.7 - 25.0) | 22 (2) | 9.1 (2.2 - 30.7) | 21 (2) | 9.5 (2.3 - 32.0) |
| Quinine | 21 (7) | 33.3 (16.5 - 55.9) | 4 (0) | 0 (0.0 - 60.2) | 0 (0) | - |
|  |  |  |  |  |  |  |
| **Total weights corrected to first day of life** | **656 (104)** | | **669 (41)** |  | **590 (43)** |  |
| **Prevalence of LBW using weights corrected to first day of life** | |  |  |  |  |  |
| No exposure | 603 (93) | 15.4 (12.6 - 18.7) | 643 (39) | 6.1 (4.4 - 8.2) | 551 (39) | 7.1 (5.1 - 9.6) |
| Artemisinin | 31 (4) | 12.9 (3.6 - 29.8) | 22 (2) | 9.1 (1.1 - 29.2) | 39 (4) | 10.3 (2.9 - 24.2) |
| Quinine | 22 (7) | 31.8 (13.9 - 54.9) | 4 (0) | 0.0 (0.0 - 60.2) | 0 (0) | - |
|  |  |  |  |  |  |  |

* 95% Exact confidence intervals

Table S3 - Small for gestation age prevalence, per exposure status and study site, ASAP cohort

|  | **Burkina Faso** | | **Mozambique** | | **Kenya** | |
| --- | --- | --- | --- | --- | --- | --- |
|  | **N (n)** | **Prevalence (95%CI) *** | **N (n)** | **Prevalence (95%CI) *** | **N (n)** | **Prevalence (95%CI) *** |
| **Total weights collected on day of birth** | **598 (117)** |  | **646 (32)** |  | **276 (69)** |  |
| **Prevalence of SGA using weights collected on day of birth** | |  |  |  |  |  |
| No exposure | 549 (111) | 20.2 (16.9 - 23.8) | 621 (31) | 5.0 (3.4 - 7.0) | 256 (61) | 23.8 (18.7 - 29.5) |
| Artemisinin | 28 (0) | 0.0 (0.0 - 12.3) | 21 (1) | 4.8 (0.1 - 23.8) | 20 (8) | 40.0 (19.1 - 64.0) |
| Quinine | 21 (6) | 28.6 (11.3 - 60.2) | 4 (0) | 0.0 (0.0 - 60.2) | 0 (0) | - |
|  |  |  |  |  |  |  |
| **Total weights corrected to first day of life** | **648 (124)** |  | **659 (33)** |  | **581 (114)** |  |
| **Prevalence of SGA using weights corrected to first day of life** | |  |  |  |  |  |
| No exposure | 595 (118) | 19.8 (16.7 - 23.3) | 634 (32) | 5.1 (3.5 - 7.1) | 543 (103) | 19.0 (15.8 - 22.5) |
| Artemisinin | 31 (0) | 0.0 (0.0 - 11.2) | 21 (1) | 4.8 (0.1 - 23.8) | 38 (11) | 29.0 (15.4 - 45.9) |
| Quinine | 22 (6) | 27.3 (10.7 - 50.2) | 4 (0) | 0.0 (0.0 - 60.2) | 0 (0) | - |
|  |  |  |  |  |  |  |

Table S4 – Meta-analysis of mean differences of birthweights (ACT-exposed versus Non-exposed), ASAP cohort

|  | **MD** | **95%CI** | **%W(fixed)** | **%W(random)** | **p-value** |
| --- | --- | --- | --- | --- | --- |
| **ACT-exposed vs Non-exposed** |  |  |  |  |  |
| ***Birthweights measured within 24 hours of birth (N)*** |  |  |  |  |  |
| Burkina Faso | 198.9 | [ 58.2; 339.7] | 45.9 | 35.8 |  |
| Mozambique | 35.7 | [-133.6; 205.1] | 31.7 | 33.4 |  |
| Kenya | -181.4 | [-382.5; 19.7] | 22.5 | 30.7 |  |
|  |  |  |  |  |  |
| Fixed effect model | 61.8 | [ -33.5; 157.1] | 100.0 |  | 0.204 |
| Random effects model | 27.5 | [-183.7; 238.6] |  | 100.00 | 0.799 |
| tau^2 = 27235.5443; H = 2.16 [1.21; 3.87]; I^2 = 78.6% [31.4%; 93.3%] | | | | | |
|  |  |  |  |  |  |
| ***Birthweights measured within 7 days of birth (N)*** |  |  |  |  |  |
| Burkina Faso | 140.5 | [ -2.4; 283.4] | 42.3 | 39.4 |  |
| Mozambique | 38.7 | [-130.6; 207.9] | 30.1 | 31.3 |  |
| Kenya | -53.0 | [-229.9; 123.8] | 27.6 | 29.3 |  |
|  |  |  |  |  |  |
| Fixed effect model | 56.4 | [-36.5; 149.3] | 100.0 |  | 0.234 |
| Random effects model | 51.9 | [-59.6; 163.4] |  | 100.00 | 0.362 |
| tau^2 = 2893.8683; H = 1.19 [1.00; 3.70]; I^2 = 29.6% [0.0%; 92.7%] | | | | | |
|  |  |  |  |  |  |

Table S5 – Meta-analysis of mean differences of birthweights (QNN-exposed versus Non-exposed), ASAP cohort

|  | **MD** | **95%CI** | **%W(fixed)** | **%W(random)** | **p-value** |
| --- | --- | --- | --- | --- | --- |
| **QNN-exposed vs Non-exposed** |  |  |  |  |  |
| ***Birthweights measured within 24 hours of birth (N)*** |  |  |  |  |  |
| Burkina Faso | -66.2 | [-283.8; 151.4] | 74.4 | 69.3 |  |
| Mozambique | 181.2 | [-190.3; 552.6] | 25.6 | 30.7 |  |
| Kenya | - | - | 0.0 | 0.0 |  |
|  |  |  |  |  |  |
| Fixed effect model | -3.0 | [-190.8; 184.8] | 100.0 |  | 0.975 |
| Random effects model | 9.8 | [-213.9; 233.5] |  | 100.00 | 0.932 |
| tau^2 = 6469.5545; H = 1.13; I^2 = 21.1% | | | | | |
|  |  |  |  |  |  |
| ***Birthweights measured within 7 days of birth (N)*** |  |  |  |  |  |
| Burkina Faso | -34.3 | [-254.3; 185.8] | 74.0 | 74.0 |  |
| Mozambique | 184.1 | [-187.3; 555.5] | 26.0 | 26.0 |  |
| Kenya | - | - | 0.0 | 0.0 |  |
|  |  |  |  |  |  |
| Fixed effect model | 22.50 | [-166.9; 211.9] | 100.0 |  | 0.816 |
| Random effects model | 22.50 | [-166.9; 211.9] |  | 100.00 | 0.816 |
| tau^2 = 0; H = 1.00; I^2 = 0.0% | | | | | |
|  |  |  |  |  |  |

Table S6 – Meta-analysis of mean differences of birthweights (QNN-exposed versus ACT-exposed), ASAP cohort

|  | **MD** | **95%CI** | **%W(fixed)** | **%W(random)** | **p-value** |
| --- | --- | --- | --- | --- | --- |
| **QNN-exposed vs ACT-exposed** |  |  |  |  |  |
| ***Birthweights measured within 24 hours of birth (N)*** |  |  |  |  |  |
| Burkina Faso | -265.1 | [-519.3; -11.0] | 71.8 | 57.7 |  |
| Mozambique | 145.5 | [-260.0; 550.9] | 28.2 | 42.3 |  |
| Kenya | - | - | 0.0 | 0.0 |  |
|  |  |  |  |  |  |
| Fixed effect model | -149.3 | [-364.7; 66.0] | 100.0 |  | 0.174 |
| Random effects model | -91.5 | [-489.0; 306.1] |  | 100.00 | 0.652 |
| tau^2 = 54479.8711; H = 1.68; I^2 = 64.6% [0.0%; 91.9%] | | | | | |
|  |  |  |  |  |  |
| ***Birthweights measured within 7 days of birth (N)*** |  |  |  |  |  |
| Burkina Faso | -174.7 | [-432.5; 83.1] | 71.2 | 62.4 |  |
| Mozambique | 145.5 | [-260.0; 550.9] | 28.8 | 37.6 |  |
| Kenya | - | - | 0.0 | 0.0 |  |
|  |  |  |  |  |  |
| Fixed effect model | -82.5 | [-300.1; 135.0] | 100.0 |  | 0.457 |
| Random effects model | -54.4 | [-358.4; 249.5] |  | 100.00 | 0.726 |
| tau^2 = 21206.9203; H = 1.31; I^2 = 41.4% | | | | | |
|  |  |  |  |  |  |

Table S7 – Meta-analysis of LBW prevalence ratio (ACT-exposed versus Non-exposed), ASAP cohort

| LBW | **PR** | **95%CI** | **%W(fixed)** | **%W(random)** | **p-value** |
| --- | --- | --- | --- | --- | --- |
| **ACT-exposed vs Non-exposed** |  |  |  |  |  |
| ***Birthweights measured within 24 hours of birth (N)*** |  |  |  |  |  |
| Burkina Faso | 0.46 | [0.12; 1.76] | 63.4 | 34.2 |  |
| Mozambique | 1.59 | [0.41; 6.19] | 18.5 | 33.8 |  |
| Kenya | 1.55 | [0.38; 6.31] | 18.1 | 32.0 |  |
|  |  |  |  |  |  |
| Fixed effect model | 0.86 | [0.39; 1.90] | 100.0 |  | 0.717 |
| Random effects model | 1.03 | [0.44; 2.41] |  | 100.00 | 0.945 |
| tau^2 = 0.0747; H = 1.07 [1.00; 3.33]; I^2 = 13.3% [0.0%; 91.0%] | | | | | |
|  |  |  |  |  |  |
| ***Birthweights measured within 7 days of birth (N)*** |  |  |  |  |  |
| Burkina Faso | 0.84 | [0.33; 2.13] | 54.0 | 41.9 |  |
| Mozambique | 1.50 | [0.38; 5.82] | 15.3 | 19.8 |  |
| Kenya | 1.45 | [0.55; 3.85] | 30.6 | 38.3 |  |
|  |  |  |  |  |  |
| Fixed effect model | 1.13 | [0.62; 2.05] | 100.0 |  | 0.700 |
| Random effects model | 1.16 | [0.63; 2.12] |  | 100.00 | 0.632 |
| tau^2 = 0; H = 1.00 [1.00; 1.98]; I^2 = 0.0% [0.0%; 74.5%] | | | | | |
|  |  |  |  |  |  |

Table S8 – Meta-analysis of LBW prevalence ratio (QNN-exposed versus Non-exposed), ASAP cohort

| LBW | **PR** | **95%CI** | **%W(fixed)** | **%W(random)** | **p-value** |
| --- | --- | --- | --- | --- | --- |
| **QNN-exposed vs Non-exposed** |  |  |  |  |  |
| ***Birthweights measured within 24 hours of birth (N)*** |  |  |  |  |  |
| Burkina Faso | 2.13 | [1.13; 4.02] | 91.7 | 94.5 |  |
| Mozambique | 1.92 | [0.14; 26.69] | 8.3 | 5.5 |  |
| Kenya | - | - | 0.0 | 0.0 |  |
|  |  |  |  |  |  |
| Fixed effect model | 2.10 | [1.13; 3.90] | 100.0 |  | 0.019 |
| Random effects model | 2.12 | [1.14; 3.93] |  | 100.00 | 0.017 |
| tau^2 = 0; H = 1.00; I^2 = 0.0% | | | | | |
|  |  |  |  |  |  |
| ***Birthweights measured within 7 days of birth (N)*** |  |  |  |  |  |
| Burkina Faso | 2.06 | [1.09; 3.91] | 91.5 | 94.4 |  |
| Mozambique | 1.81 | [0.13; 25.13] | 8.5 | 5.6 |  |
| Kenya | - | - | 0.0 | 0.0 |  |
|  |  |  |  |  |  |
| Fixed effect model | 2.03 | [1.09; 3.78] | 100.0 |  | 0.027 |
| Random effects model | 2.05 | [1.10; 3.81] |  | 100.00 | 0.024 |
| tau^2 = 0; H = 1.00; I^2 = 0.0% | | | | | |
|  |  |  |  |  |  |

Table S9 – Meta-analysis of LBW prevalence ratio (QNN-exposed versus ACT-exposed), ASAP cohort

| LBW | **PR** | **95%CI** | **%W(fixed)** | **%W(random)** | **p-value** |
| --- | --- | --- | --- | --- | --- |
| **QNN-exposed vs ACT-exposed** |  |  |  |  |  |
| ***Birthweights measured within 24 hours of birth (N)*** |  |  |  |  |  |
| Burkina Faso | 4.67 | [1.08; 20.23] | 65.8 | 79.2 |  |
| Mozambique | 1.00 | [0.06; 17.51] | 34.2 | 20.8 |  |
| Kenya | - | - | 0.0 | 0.0 |  |
|  |  |  |  |  |  |
| Fixed effect model | 3.38 | [0.98; 11.73] | 100.0 |  | 0.055 |
| Random effects model | 3.39 | [0.92; 12.49] |  | 100.00 | 0.067 |
| tau^2 = 0; H = 1.00; I^2 = 0.0% | | | | | |
|  |  |  |  |  |  |
| ***Birthweights measured within 7 days of birth (N)*** |  |  |  |  |  |
| Burkina Faso | 2.47 | [0.82; 7.41] | 78.8 | 87.1 |  |
| Mozambique | 1.00 | [0.06; 17.51] | 21.2 | 12.9 |  |
| Kenya | - | - | 0.0 | 0.0 |  |
|  |  |  |  |  |  |
| Fixed effect model | 2.14 | [0.78; 5.89] | 100.0 |  | 0.142 |
| Random effects model | 2.20 | [0.79; 6.13] |  | 100.00 | 0.133 |
| tau^2 = 0; H = 1.00; I^2 = 0.0% | | | | | |
|  |  |  |  |  |  |

Table S10 – Meta-analysis of SGA prevalence ratio (ACT-exposed versus Non-exposed), ASAP cohort

| SGA | **PR** | **95%CI** | **%W(fixed)** | **%W(random)** | **p-value** |
| --- | --- | --- | --- | --- | --- |
| **ACT-exposed vs Non-exposed** |  |  |  |  |  |
| ***Birthweights measured within 24 hours of birth (N)*** |  |  |  |  |  |
| Burkina Faso | 0.09 | [0.01; 1.36] | 50.7 | 24.8 |  |
| Mozambique | 0.95 | [0.14; 6.66] | 9.2 | 31.9 |  |
| Kenya | 1.68 | [0.94; 3.00] | 40.1 | 43.3 |  |
|  |  |  |  |  |  |
| Fixed effect model | 0.80 | [0.44; 1.46] | 100.0 |  | 0.475 |
| Random effects model | 0.67 | [0.09; 5.21] |  | 100.00 | 0.704 |
| tau^2 = 2.4336; H = 2.09 [1.16; 3.77]; I^2 = 77.1% [25.5%; 93.0%] | | | | | |
|  |  |  |  |  |  |
| ***Birthweights measured within 7 days of birth (N)*** |  |  |  |  |  |
| Burkina Faso | 0.08 | [0.01; 1.25] | 43.8 | 23.5 |  |
| Mozambique | 0.94 | [0.14; 6.58] | 7.4 | 31.3 |  |
| Kenya | 1.53 | [0.90; 2.59] | 48.8 | 45.2 |  |
|  |  |  |  |  |  |
| Fixed effect model | 0.85 | [0.50; 1.44] | 100.0 |  | 0.543 |
| Random effects model | 0.66 | [0.10; 4.34] |  | 100.00 | 0.663 |
| tau^2 = 1.9840; H = 1.95 [1.06; 3.56]; I^2 = 73.6% [11.7%; 92.1%] | | | | | |
|  |  |  |  |  |  |

Table S11 – Meta-analysis of SGA prevalence ratio (QNN-exposed versus Non-exposed), ASAP cohort

| SGA | **PR** | **95%CI** | **%W(fixed)** | **%W(random)** | **p-value** |
| --- | --- | --- | --- | --- | --- |
| **QNN-exposed vs Non-exposed** |  |  |  |  |  |
| ***Birthweights measured within 24 hours of birth (N)*** |  |  |  |  |  |
| Burkina Faso | 1.41 | [0.70; 2.84] | 94.2 | 93.5 |  |
| Mozambique | 2.19 | [0.16; 30.58] | 5.8 | 6.5 |  |
| Kenya | - | - | 0.0 | 0.0 |  |
|  |  |  |  |  |  |
| Fixed effect model | 1.45 | [0.74; 2.83] | 100.0 |  | 0.283 |
| Random effects model | 1.45 | [0.74; 2.85] |  | 100.00 | 0.276 |
| tau^2 = 0; H = 1.00; I^2 = 0.0% | | | | | |
|  |  |  |  |  |  |
| ***Birthweights measured within 7 days of birth (N)*** |  |  |  |  |  |
| Burkina Faso | 1.38 | [0.68; 2.77] | 94.3 | 93.4 |  |
| Mozambique | 2.17 | [0.16; 30.24] | 5.7 | 6.6 |  |
| Kenya | - | - | 0.0 | 0.0 |  |
|  |  |  |  |  |  |
| Fixed effect model | 1.41 | [0.72; 2.77] | 100.0 |  | 0.322 |
| Random effects model | 1.42 | [0.72; 2.79] |  | 100.00 | 0.313 |
| tau^2 = 0; H = 1.00; I^2 = 0.0% | | | | | |
|  |  |  |  |  |  |

Table S12 – Meta-analysis of SGA prevalence ratio (QNN-exposed versus ACT-exposed), ASAP cohort

| SGA | **PR** | **95%CI** | **%W(fixed)** | **%W(random)** | **p-value** |
| --- | --- | --- | --- | --- | --- |
| **QNN-exposed vs ACT-exposed** |  |  |  |  |  |
| ***Birthweights measured within 24 hours of birth (N)*** |  |  |  |  |  |
| Burkina Faso | 17.23 | [1.03; 289.52] | 43.7 | 52.6 |  |
| Mozambique | 1.59 | [0.08; 33.13] | 56.3 | 47.4 |  |
| Kenya | - | - | 0.0 | 0.0 |  |
|  |  |  |  |  |  |
| Fixed effect model | 8.32 | [1.23; 56.28] | 100.0 |  | 0.030 |
| Random effects model | 5.57 | [0.48; 64.23] |  | 100.00 | 0.168 |
| tau^2 = 0.8837; H = 1.18; I^2 = 28.3% | | | | | |
|  |  |  |  |  |  |
| ***Birthweights measured within 7 days of birth (N)*** |  |  |  |  |  |
| Burkina Faso | 18.20 | [1.08; 306.90] | 42.9 | 52.5 |  |
| Mozambique | 1.59 | [0.08; 33.13] | 57.1 | 47.5 |  |
| Kenya | - | - | 0.0 | 0.0 |  |
|  |  |  |  |  |  |
| Fixed effect model | 8.60 | [1.29; 57.57] | 100.0 |  | 0.027 |
| Random effects model | 5.72 | [0.47; 69.59] |  | 100.00 | 0.172 |
| tau^2 = 1.0223; H = 1.21; I^2 = 31.4% | | | | | |
|  |  |  |  |  |  |

Table S13 – Meta-analysis of prematurity prevalence ratio (ACT-exposed versus Non-exposed), ASAP cohort

|  | **PR** | **95%CI** | **%W(fixed)** | **%W(random)** | **p-value** |
| --- | --- | --- | --- | --- | --- |
| **ACT-exposed vs Non-exposed** |  |  |  |  |  |
| ***Birthweights measured within 24 hours of birth (N)*** |  |  |  |  |  |
| Burkina Faso | 2.56 | [0.97; 6.76] | 37.9 | 52.9 |  |
| Mozambique | 0.45 | [0.07; 3.11] | 54.5 | 25.4 |  |
| Kenya | 3.11 | [0.36; 26.56] | 7.6 | 21.8 |  |
|  |  |  |  |  |  |
| Fixed effect model | 1.45 | [0.65; 3.23] | 100.0 |  | 0.360 |
| Random effects model | 1.72 | [0.54; 5.51] |  | 100.00 | 0.361 |
| tau^2 = 0.4229; H = 1.27 [1.00; 2.26]; I^2 = 37.6% [0.0%; 80.4%] | | | | | |
|  |  |  |  |  |  |
| ***Birthweights measured within 7 days of birth (N)*** |  |  |  |  |  |
| Burkina Faso | 2.63 | [1.11; 6.22] | 35.3 | 49.9 |  |
| Mozambique | 0.45 | [0.07; 3.12] | 41.5 | 25.5 |  |
| Kenya | 0.78 | [0.11; 5.73] | 23.2 | 24.6 |  |
|  |  |  |  |  |  |
| Fixed effect model | 1.30 | [0.62; 2.71] | 100.0 |  | 0.487 |
| Random effects model | 1.25 | [0.36; 4.34] |  | 100.00 | 0.729 |
| tau^2 = 0.6199; H = 1.41 [1.00; 2.61]; I^2 = 49.6% [0.0%; 85.4%] | | | | | |
|  |  |  |  |  |  |

Table S14 – Meta-analysis of prematurity prevalence ratio (QNN-exposed versus Non-exposed), ASAP cohort

|  | **PR** | **95%CI** | **%W(fixed)** | **%W(random)** | **p-value** |
| --- | --- | --- | --- | --- | --- |
| **QNN-exposed vs Non-exposed** |  |  |  |  |  |
| ***Birthweights measured within 24 hours of birth (N)*** |  |  |  |  |  |
| Burkina Faso | 0.85 | [0.12; 5.96] | 69.2 | 64.6 |  |
| Mozambique | 1.10 | [0.08; 15.11] | 30.8 | 35.4 |  |
| Kenya | - | - | 0.0 | 0.0 |  |
|  |  |  |  |  |  |
| Fixed effect model | 0.90 | [0.19; 4.29] | 100.0 |  | 0.890 |
| Random effects model | 0.93 | [0.20; 4.45] |  | 100.00 | 0.931 |
| tau^2 = 0; H = 1.00; I^2 = 0.0% | | | | | |
|  |  |  |  |  |  |
| ***Birthweights measured within 7 days of birth (N)*** |  |  |  |  |  |
| Burkina Faso | 0.74 | [0.11; 5.16] | 72.3 | 64.6 |  |
| Mozambique | 1.10 | [0.08; 15.19] | 27.7 | 35.4 |  |
| Kenya | - | - | 0.0 | 0.0 |  |
|  |  |  |  |  |  |
| Fixed effect model | 0.81 | [0.17; 3.88] | 100.0 |  | 0.793 |
| Random effects model | 0.85 | [0.19; 4.06] |  | 100.00 | 0.841 |
| tau^2 = 0; H = 1.00; I^2 = 0.0% | | | | | |
|  |  |  |  |  |  |

Table S15 – Meta-analysis of prematurity prevalence ratio (QNN-exposed versus ACT-exposed), ASAP cohort

|  | **PR** | **95%CI** | **%W(fixed)** | **%W(random)** | **p-value** |
| --- | --- | --- | --- | --- | --- |
| **QNN-exposed vs ACT-exposed** |  |  |  |  |  |
| ***Birthweights measured within 24 hours of birth (N)*** |  |  |  |  |  |
| Burkina Faso | 0.33 | [0.04; 2.77] | 86.5 | 67.3 |  |
| Mozambique | 1.67 | [0.08; 34.72] | 13.5 | 32.7 |  |
| Kenya | - | - | 0.0 | 0.0 |  |
|  |  |  |  |  |  |
| Fixed effect model | 0.50 | [0.09; 2.63] | 100.0 |  | 0.409 |
| Random effects model | 0.56 | [0.10; 3.20] |  | 100.00 | 0.518 |
| tau^2 = 0; H = 1.00; I^2 = 0.0% | | | | | |
|  |  |  |  |  |  |
| ***Birthweights measured within 7 days of birth (N)*** |  |  |  |  |  |
| Burkina Faso | 0.28 | [0.04; 2.25] | 88.6 | 68.1 |  |
| Mozambique | 1.67 | [0.08; 34.72] | 11.4 | 31.9 |  |
| Kenya | - | - | 0.0 | 0.0 |  |
|  |  |  |  |  |  |
| Fixed effect model | 0.42 | [0.08; 2.19] | 100.0 |  | 0.306 |
| Random effects model | 0.50 | [0.09; 2.76] |  | 100.00 | 0.423 |
| tau^2 = 0; H = 1.00; I^2 = 0.0% | | | | | |
|  |  |  |  |  |  |

Table S16 - Adjusted associations for prematurity prevalence

|  | **Prevalence-Ratio (95%CI**^†^**)** | | | |
| --- | --- | --- | --- | --- |
|  | **Unadjusted*** | **p-value** | **Adjusted**** | **p-value** |
| **Birthweights measured within 24 hours of birth (N)** | 1539 |  | 1533 |  |
| No exposure | 1 (reference) |  | 1 (reference) |  |
| Artemisinin | 1.32 (0.53 - 2.65) | 0.504 | 1.35 (0.55 - 2.66) | 0.461 |
| Quinine | 0.42 (0.02 - 2.13) | 0.379 | 0.37 (0.01 - 1.82) | 0.287 |
|  |  |  |  |  |
| **Birthweights measured within 7 days of birth (N)** | 1911 |  | 1905 |  |
| No exposure | 1 (reference) |  | 1 (reference) |  |
| Artemisinin | 1.22 (0.53 - 2.33) | 0.610 | 1.22 (0.53 - 2.34) | 0.610 |
| Quinine | 0.37 (0.01 - 1.90) | 0.309 | 0.34 (0.01 - 1.80) | 0.269 |
|  |  |  |  |  |
| *Quinine vs Artemisinin* |  |  |  |  |
| Artemisinin | 1 (reference) |  | 1 (reference) |  |
| Quinine with no Kenya data (N = 79) | 0.20 (0.01 - 1.27) | 0.095 | 0.15 (0.00 - 1.23) | 0.089 |
| Quinine with Kenya data (N = 118) | 0.20 (0.01 - 1.32) | 0.102 | 0.25 (0.01 - 1.71) | 0.186 |
|  |  |  |  |  |

* Unadjusted regression includes dummy indicators for site

**Adjusted for site, age at recruitment, gravidity, marital status and education level

^†^95%CI - 95% Credible Interval based on posterior distribution replication

## References

1. Villar J, Cheikh Ismail L, Victora CG, Ohuma EO, Bertino E, Altman DG, Lambert A, Papageorghiou AT, Carvalho M, Jaffer YA, et al: **International standards for newborn weight, length, and head circumference by gestational age and sex: the Newborn Cross-Sectional Study of the INTERGROWTH-21st Project.** *Lancet* 2014, **384:**857-868.
